# Nationwide epidemiology of HPV E6/E7 mRNA in China: genotype distribution in 11,118 women

**DOI:** 10.3389/fpubh.2026.1797051

**Published:** 2026-04-24

**Authors:** Shengyang Xu, Yan Li, Xunfei Yi, Zhanli Yan, Tiantian Zeng, Ning Meng, Jingna Sun

**Affiliations:** 1 Dian Diagnostics Group Co., Ltd., Hangzhou, China; 2 Department of Gynecology, Tianjin First Central Hospital, Tianjin, China; 3 Guangzhou Biotron Biotechnology Co., Ltd., Guangzhou, China; 4 Hebei Innovation Center of Clinical Medical Laboratory Technology, Department of Clinical Laboratory, The First Hospital of Hebei Medical University, Shijiazhuang, China

**Keywords:** cervical cancer, China, epidemiology, genotype distribution, HPV E6/E7 mRNA, human papillomavirus

## Abstract

**Background:**

Persistent infection with carcinogenic human papillomavirus (HPV) is the necessary cause of cervical cancer. However, the distribution of transcriptionally active high-risk HPV genotypes across age groups and geographic regions in China remains insufficiently characterized. We aimed to define the nationwide epidemiologic profile of HPV E6/E7 mRNA positivity in China and to evaluate age-, region-, and proxy-defined source-population heterogeneity.

**Methods:**

In this retrospective cross-sectional study, 11,118 female cervical specimens retrieved for HPV E6/E7 mRNA testing between August 14, 2024 and October 23, 2025 were analyzed from seven geographic regions of China. Fourteen high-risk HPV genotypes were detected using the Biotron HPV E6/E7 mRNA Genotyping Assay. Descriptive, co-detection, and multivariable logistic regression analyses with institution-level cluster-robust standard errors were performed.

**Results:**

Overall high-risk HPV E6/E7 mRNA positivity was 16.32% (1,815/11,118). HPV52, HPV58, and HPV16 were the dominant genotypes, followed by HPV18 and HPV51. Overall positivity varied significantly across age groups, regions, institution-level proxy categories, and department-based clinical-context proxy categories. Central China had the highest overall positivity rate, whereas East China had the lowest. Significant age-related heterogeneity was observed for several major genotypes, especially HPV52, HPV58, and HPV16. Multi-type positivity accounted for 11.8% of positive samples, with HPV52 and HPV58 forming the most prominent co-detection pattern. In multivariable analyses, older age and several non-East regions were associated with higher odds of overall positivity, whereas the opportunistic screening proxy group showed markedly lower odds of positivity than the gynecology-related clinical attendance proxy group.

**Conclusion:**

Transcriptionally active high-risk HPV infection in China is characterized by dominance of HPV52, HPV58, and HPV16 and by marked heterogeneity across age, geography, and proxy-defined testing contexts. These findings may support more refined HPV surveillance and risk-adapted prevention strategies.

## Introduction

Persistent infection with high-risk human papillomavirus (HPV) is the necessary cause of the vast majority of cervical cancer cases (1–3). Recent global syntheses have further quantified genotype-specific carcinogenicity, showing that HPV16 and HPV18 account for the largest shares of the worldwide attributable fraction, while other genotypes, including HPV45, HPV33, HPV58, HPV31, and HPV52, contribute a substantial residual burden (4–6). Importantly, genotype-specific attributable fractions vary across regions, with different contributions of non-16/18 genotypes observed in Asia, Africa, and other settings (4, 7). This genotype-level heterogeneity has important implications for cervical cancer prevention and supports movement toward more context-specific screening and vaccination strategies (2).

As the main contributor to the global cancer burden, in China, prevention and control are characterized by a distinct genotype spectrum that diverges from patterns typically reported in Europe and North America. Large DNA-based epidemiological studies show that, beyond HPV16/18, HPV52 and HPV58 are disproportionately prevalent in Chinese women, together with frequent detection of HPV39, 51, and 53; these features have been consistently observed in megacities such as Beijing and Guangzhou (8–10) and in other regions, including Huizhou, Yueyang, and Hengyang (11–14). Age-specific prevalence often follows a U-shaped curve, suggesting region- and age-dependent infection dynamics and potential differences in progression risk (14, 15). Collectively, these DNA-level data provide critical baselines for primary and secondary prevention in China.

However, prevailing evidence largely relies on HPV DNA testing, which—despite high analytical sensitivity—cannot distinguish transient infections from truly oncogenic, persistent infections because it does not directly assess transcriptional activation of the viral E6/E7 oncogenes (1, 2). By contrast, HPV E6/E7 mRNA detection provides a direct measure of viral transforming activity and offers greater clinical specificity for identifying infections at higher risk of progressing to high-grade lesions. As such, mRNA-based assays hold considerable promise for refining risk stratification among HPV-positive individuals, curbing unnecessary colposcopy and excisional treatment, and improving screening efficiency. Despite extensive DNA-based epidemiology in China, there is no nationwide surveillance using E6/E7 mRNA endpoints; consequently, we lack clarity on which regions and genotypes are transcriptionally active and pose the greatest immediate threat—an evidence gap that constrains precision prevention.

To address this gap, we conducted a large-scale retrospective study of HPV E6/E7 mRNA positivity across seven major geographic regions of China, covering 14 high-risk genotypes. In addition to describing national, regional, and age-specific genotype distributions, we incorporated proxy indicators of submitting-institution level and clinical testing context to better characterize source-population heterogeneity in this real-world testing cohort (16).

## Materials and methods

### Study design and population

**Figure 1:**
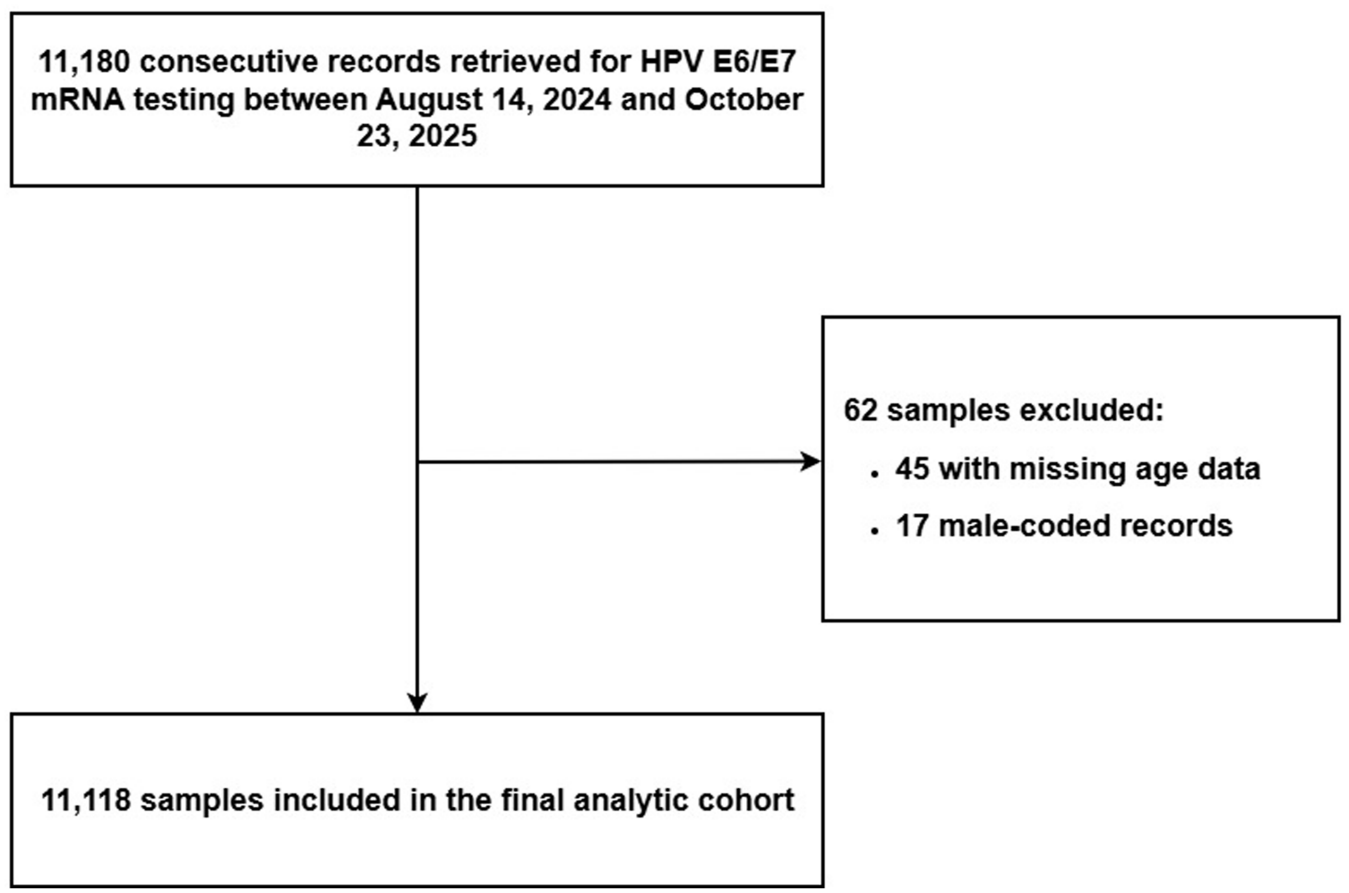
Flowchart of study sample selection. A total of 11,180 consecutive records retrieved for HPV E6/E7 mRNA testing between August 14, 2024, and October 23, 2025, were reviewed. After exclusion of 45 records with missing or non-interpretable age data and 17 male-coded records, 11,118 female cervical specimens were included in the final analytic cohort. Institution-level proxy information was classifiable for 10,876 samples, and department-based clinical-context proxy information was classifiable for 10,608 samples.

We conducted a retrospective, cross-sectional analysis of consecutive records retrieved for HPV E6/E7 mRNA testing between August 14, 2024 and October 23, 2025 from participating institutions across seven geographic regions of China: East, Northwest, Southwest, South, Northeast, North, and Central China. As shown in Figure 1, a total of 11,180 records were reviewed. After exclusion of 45 records with missing or non-interpretable age data and 17 male-coded records, 11,118 female cervical specimens were included in the final analytic cohort. Age was analyzed both as a continuous variable and as predefined categorical strata (≤30, 31–40, 41–50, 51–60, and >60 years), depending on the analytic context. Baseline characteristics of the analytic cohort are summarized in Table 1. This study used de-identified laboratory data generated during routine clinical testing. The study was conducted in accordance with the Declaration of Helsinki. According to local requirements for retrospective analyses of de-identified data, formal ethical review and informed consent were not required.

**Table 1:** Baseline demographic characteristics and source-population proxy variables of the analytic cohort.

Characteristics	Overall cohort
Age, median [IQR], years	42.00 [35.00–51.00]
Region, *n*/*N* (%)	
East	4.926/11.118 (44.3)
Northwest	1.157/11.118 (10.4)
Southwest	2.863/11.118 (25.8)
South	399/11.118 (3.6)
Northeast	358/11.118 (3.2)
North	794/11.118 (7.1)
Central	621/11.118 (5.6)
Institution-level proxy, *n*/*N* (%)	
Municipal/provincial	6.096/10.876 (56.1)
County/community	4.780/10.876 (43.9)
Department-based proxy, *n*/*N* (%)	
Gynecology-related	6.654/10.608 (62.7)
Opportunistic screening	3.954/10.608 (37.3)

Data are presented as median (interquartile range, IQR) for age and as *n* (%) for categorical variables. Percentages were calculated using the number of classifiable records within each variable as the denominator. Region was available for all 11,118 participants. Institution-level proxy was classifiable for 10,876 participants, and department-based clinical-context proxy was classifiable for 10,608 participants. The institution-level proxy was derived from the submitting institution and used to approximate the administrative level and service setting of the submitting site rather than the participant’s confirmed place of residence. The department-based clinical-context proxy was derived from the submitting department and used to approximate the testing context, including gynecology-related clinical attendance versus opportunistic screening. IQR, interquartile range.

### Definition of analytic variables and proxy classifications

The primary outcome was any high-risk HPV E6/E7 mRNA positivity, defined as detection of at least one of the 14 targeted high-risk HPV genotypes. Genotype-specific positivity was additionally evaluated for HPV16, HPV52, and HPV58, which were the most prevalent transcriptionally active genotypes in the cohort. Multiple-type infection was defined as concurrent detection of two or more HPV genotypes in the same specimen.

To better characterize source-population heterogeneity and clinical testing context, two proxy variables were derived from routinely available laboratory submission information. First, based on the name and administrative level of the submitting institution, samples were categorized into a provincial/municipal-level institution proxy or a county/community institution proxy. Records that could not be confidently classified were treated as unclassifiable for analyses involving this variable. This proxy was used to approximate the administrative level and service setting of the submitting institution rather than the participant’s confirmed place of residence. Second, based on the submitting department, samples were categorized into a gynecology-related clinical attendance proxy or an opportunistic screening proxy. The gynecology-related category included gynecology, obstetrics and gynecology, and gynecology outpatient departments. The opportunistic screening category included maternal and child health, health examination, and health management settings. Records that could not be confidently assigned were treated as unclassifiable for analyses involving this variable.

This proxy was used to approximate clinical testing context rather than the definitive indication for testing. Institution-level proxy information was classifiable for 10,876 samples, and department-based clinical-context proxy information was classifiable for 10,608 samples.

### Specimen collection, transport and laboratory testing

Cervical exfoliated cell samples were collected using a cervical brush after removal of excessive cervical secretions and were placed into cell preservation medium. According to the standard operating procedures of the central laboratory, specimens were maintained under cold-chain conditions after collection, stored at 2–8 °C when short-term holding was required, and transported to Hangzhou Dian Medical Testing Center in insulated containers with dry ice. Samples were generally processed within 24–72 h after collection, and repeated freeze–thaw cycles were avoided. HPV E6/E7 mRNA genotyping was performed using the Biotron HPV E6/E7 mRNA Genotyping Assay (Guangzhou Biotron Technology Co., Ltd., Guangzhou, China) on a SLAN-96S real-time PCR system (Hongshi, Shanghai, China). The assay targets 14 high-risk or probably high-risk genotypes: HPV16, 18, 31, 33, 35, 39, 45, 51, 52, 56, 58, 59, 66, and 68. The analytical principle of the assay and genotype-specific detection design are summarized in Supplementary Table S1. The assay consists of reverse transcription followed by multiplex fluorescence PCR and melting-curve analysis for genotype identification. Signal acquisition was performed in the FAM, HEX, ROX, and CY5 channels. Final genotype calls were generated using the manufacturer’s companion analysis software based on channel-specific amplification signals together with predefined melting temperature (Tm) profiles and peak-height change thresholds (Supplementary Table S2).

### Assay interpretation and quality control

A sample was considered positive for HPV E6/E7 mRNA if amplification was detected with a Ct value ≤40 in any of the analytical channels (FAM, HEX, or ROX), with genotype assignment determined according to the corresponding Tm peak pattern by the analysis software. Samples with no amplification in FAM, HEX, and ROX were considered negative only when the endogenous human internal control in the CY5 channel showed a valid amplification curve with Ct ≤ 35. This internal control was used to confirm adequate cellular sampling, successful RNA extraction, and effective reverse transcription. Samples with invalid internal-control results were considered uninterpretable and were repeated according to laboratory procedures.

Each run included positive and negative controls. The negative control was required to show no valid amplification in FAM, HEX, and ROX together with a valid internal-control signal in CY5. The positive control was required to generate valid amplification signals and the expected genotype calls.

According to the manufacturer’s performance documentation, the assay has a limit of detection of no more than 100 copies/reaction. Reported analytical precision was <5% across within-run, between-run, between-operator, between-instrument, and between-laboratory evaluations. No evident cross-reactivity was observed with non-target HPV genotypes or common genital tract pathogens. In simulated co-detection conditions, a high-concentration target (10,000 copies/reaction) did not interfere with detection of a low-concentration target (100 copies/reaction), supporting the assay’s analytical performance in multi-type samples.

### Co-detection network analysis

To characterize co-detection patterns among transcriptionally active HPV genotypes, analyses were performed among HPV E6/E7 mRNA-positive samples using R version 4.3.1. Detection patterns were first summarized as single-type, double-type, triple-type, and quadruple-or-more type positivity.

An undirected weighted co-detection network was then constructed using the igraph package (version 2.1.1) as a descriptive visualization of pairwise genotype co-occurrence. In this network, nodes represent HPV genotypes and edges represent observed pairwise co-detection frequencies across multi-type positive samples. Node size was scaled according to degree, defined as the number of distinct genotypes with which a given genotype co-occurred at least once. Edge thickness was proportional to pairwise co-detection frequency. This network was intended as a descriptive representation of co-detection structure rather than a formal causal, mechanistic, or synergistic model. Complementary to the network analysis, pairwise co-occurrence frequencies were visualized via heatmaps. For specific dual-detection analysis, exclusive double-detection pairs (excluding triple or higher-order combinations) were calculated and ranked to identify the most prevalent pairwise interactions.

### Statistical analysis

Descriptive analyses summarized overall and genotype-specific HPV E6/E7 mRNA positivity by age group, geographic region, institution-level proxy category, and department-based clinical-context proxy category. Baseline characteristics are shown in Table 1, overall positivity patterns in Table 2, age-specific genotype distributions in Table 3, regional genotype distributions in Table 4, institution-level proxy comparisons in Table 5, and department-based proxy comparisons in Table 6. Prevalence estimates are presented as proportions with 95% confidence intervals (CIs).

**Table 2:** Prevalence of any high-risk HPV E6/E7 mRNA positivity according to demographic and source-population proxy characteristics.

Characteristics	Positive, *n*/*N* (%)	*p*-value
Age group		<0.001
≤30	236/1,460 (16.2)	
31–40	507/3,527 (14.4)	
41–50	449/3,210 (14.0)	
51–60	430/2,132 (20.2)	
>60	193/789 (24.5)	
Region		<0.001
East	638/4,926 (13.0)	
Northwest	167/1,157 (14.4)	
Southwest	479/2,863 (16.7)	
South	98/399 (24.6)	
Northeast	100/358 (27.9)	
North	148/794 (18.6)	
Central	185/621 (29.8)	
Institution-level proxy		<0.001
Provincial/municipal	912/6,096 (15.0)	
County/community	844/4,780 (17.7)	
Department-based proxy		<0.001
Gynecology-related	1,339/6,654 (20.1)	
Opportunistic screening	340/3,954 (8.6)	

Data are presented as *n*/*N* (%), where *n* denotes the number of participants positive for any of the 14 high-risk HPV E6/E7 mRNA genotypes and *N* denotes the total number of classifiable participants within each subgroup. *p*-values were derived from Pearson’s Chi-square tests assessing overall heterogeneity in HPV positivity across categories within each variable.

**Table 3:** Age-specific prevalence of individual high-risk HPV E6/E7 mRNA genotypes.

High-risk HPV genotype	Overall positive, *n* (%)	95% CI	≤30, *n* (%), (*n* = 1,460)	31–40, *n* (%), (*n* = 3,527)	41–50, *n* (%), (*n* = 3,210)	51–60, *n* (%), (*n* = 2,132)	>60, *n* (%), (*n* = 789)	*p*-value
HPV52	653 (5.87)	5.45–6.33	72 (4.93)	197 (5.59)	168 (5.23)	155 (7.27)	61 (7.73)	0.0014
HPV58	308 (2.77)	2.48–3.10	36 (2.47)	74 (2.10)	73 (2.27)	79 (3.71)	46 (5.83)	<0.001
HPV16	238 (2.14)	1.88–2.43	43 (2.95)	60 (1.70)	52 (1.62)	54 (2.53)	29 (3.68)	<0.001
HPV18	133 (1.20)	1.01–1.42	27 (1.85)	35 (0.99)	30 (0.93)	30 (1.41)	11 (1.39)	0.0512
HPV51	116 (1.04)	0.87–1.25	15 (1.03)	42 (1.19)	25 (0.78)	23 (1.08)	11 (1.39)	0.4213
HPV33	109 (0.98)	0.81–1.19	12 (0.82)	32 (0.91)	24 (0.75)	24 (1.13)	17 (2.15)	0.0072
HPV56	108 (0.97)	0.80–1.18	14 (0.96)	24 (0.68)	22 (0.69)	28 (1.31)	20 (2.53)	<0.001
HPV68	101 (0.91)	0.74–1.11	18 (1.23)	25 (0.71)	28 (0.87)	26 (1.22)	4 (0.51)	0.1349
HPV31	78 (0.70)	0.56–0.88	12 (0.82)	14 (0.40)	19 (0.59)	20 (0.94)	13 (1.65)	0.0016
HPV39	71 (0.64)	0.50–0.81	8 (0.55)	25 (0.71)	15 (0.47)	14 (0.66)	9 (1.14)	0.2779
HPV35	56 (0.50)	0.38–0.66	6 (0.41)	11 (0.31)	11 (0.34)	21 (0.98)	7 (0.89)	0.0050^a^
HPV59	39 (0.35)	0.25–0.48	8 (0.55)	10 (0.28)	7 (0.22)	10 (0.47)	4 (0.51)	0.2239^a^
HPV45	28 (0.25)	0.17–0.37	7 (0.48)	5 (0.14)	10 (0.31)	3 (0.14)	3 (0.38)	0.1229^a^
HPV66	20 (0.18)	0.11–0.28	1 (0.07)	8 (0.23)	5 (0.16)	5 (0.23)	1 (0.13)	0.7836^a^

Data are shown as *n* (%). Overall prevalence was calculated using the full analytic cohort (*N* = 11,118), and age-specific prevalence was calculated within each age group. Genotype-specific categories were not mutually exclusive because participants with co-detections could be counted in more than one HPV genotype category.

HPV, human papillomavirus; CI, confidence interval.

a *p*-values were calculated using Fisher’s exact test; all others were calculated using Pearson’s Chi-square test.

**Table 4:** Region-specific prevalence of individual high-risk HPV E6/E7 mRNA genotypes across China.

High-risk HPV genotype	Overall positive, *n* (%)	95% CI	East, *n* (%) (*n* = 4,926)	Northwest, *n* (%), (*n* = 1,157)	Southwest, *n* (%), (*n* = 2,863)	South, *n* (%), (*n* = 399)	Northeast, *n* (%), (*n* = 358)	North, *n* (%), (*n* = 794)	Central, *n* (%), (*n* = 621)	*p*-value
HPV52	653 (5.87)	5.45–6.33	239 (4.85)	49 (4.24)	182 (6.36)	35 (8.77)	34 (9.50)	44 (5.54)	70 (11.27)	<0.001^a^
HPV58	308 (2.77)	2.48–3.10	122 (2.48)	24 (2.07)	79 (2.76)	19 (4.76)	15 (4.19)	24 (3.02)	25 (4.03)	0.0177^a^
HPV16	238 (2.14)	1.88–2.43	45 (0.91)	30 (2.59)	87 (3.04)	11 (2.76)	16 (4.47)	25 (3.15)	24 (3.86)	<0.001^a^
HPV18	133 (1.20)	1.01–1.42	34 (0.69)	11 (0.95)	42 (1.47)	10 (2.51)	14 (3.91)	9 (1.13)	13 (2.09)	<0.001
HPV51	116 (1.04)	0.87–1.25	38 (0.77)	11 (0.95)	29 (1.01)	10 (2.51)	3 (0.84)	12 (1.51)	13 (2.09)	0.0040
HPV33	109 (0.98)	0.81–1.19	40 (0.81)	8 (0.69)	38 (1.33)	2 (0.50)	4 (1.12)	4 (0.50)	13 (2.09)	0.0230
HPV56	108 (0.97)	0.80–1.18	37 (0.75)	13 (1.12)	30 (1.05)	2 (0.50)	7 (1.96)	5 (0.63)	14 (2.25)	0.0145
HPV68	101 (0.91)	0.74–1.11	43 (0.87)	9 (0.78)	19 (0.66)	6 (1.50)	4 (1.12)	10 (1.26)	10 (1.61)	0.1914
HPV31	78 (0.70)	0.56–0.88	30 (0.61)	4 (0.35)	12 (0.42)	5 (1.25)	9 (2.51)	10 (1.26)	8 (1.29)	<0.001
HPV39	71 (0.64)	0.50–0.81	23 (0.47)	8 (0.69)	19 (0.66)	1 (0.25)	2 (0.56)	8 (1.01)	10 (1.61)	0.0625
HPV35	56 (0.50)	0.38–0.66	19 (0.39)	7 (0.61)	13 (0.45)	1 (0.25)	5 (1.40)	8 (1.01)	3 (0.48)	0.0585
HPV59	39 (0.35)	0.25–0.48	15 (0.30)	5 (0.43)	8 (0.28)	2 (0.50)	3 (0.84)	2 (0.25)	4 (0.64)	0.3513
HPV45	28 (0.25)	0.17–0.37	10 (0.20)	1 (0.09)	10 (0.35)	1 (0.25)	2 (0.56)	3 (0.38)	1 (0.16)	0.5557
HPV66	20 (0.18)	0.11–0.28	4 (0.08)	2 (0.17)	6 (0.21)	2 (0.50)	3 (0.84)	2 (0.25)	1 (0.16)	0.0270

Values are presented as *n* (%). “Overall positive” refers to the number and proportion of participants positive for the corresponding HPV genotype in the full analytic cohort (*N* = 11,118). Regional percentages were calculated using the total number of participants within each region as the denominator. Genotype-specific positivity was not mutually exclusive; therefore, participants with multiple detections could contribute to more than one genotype category. The 95% CIs were calculated for the overall prevalence of each genotype. *p*-values were used to assess overall geographic heterogeneity in genotype-specific positivity across the seven regions.

HPV, human papillomavirus; CI, confidence interval.

a *p*-values were calculated using Pearson’s Chi-square test; all others were calculated using Fisher’s exact test.

**Table 5:** Institution-level differences in the prevalence of individual high-risk HPV E6/E7 mRNA genotypes.

High-risk HPV genotype	Overall positive, *n* (%)	95% CI	Provincial/municipal, *n* (%), (*n* = 6,096)	County/community, *n* (%), (*n* = 4,780)	*p*-value
HPV52	634 (5.83)	5.40–6.29	328 (5.38)	306 (6.40)	0.0268
HPV58	297 (2.73)	2.44–3.06	156 (2.56)	141 (2.95)	0.2373
HPV16	232 (2.13)	1.87–2.43	111 (1.82)	121 (2.53)	0.0132
HPV18	130 (1.20)	1.00–1.42	67 (1.10)	63 (1.32)	0.3402
HPV51	110 (1.01)	0.84–1.22	55 (0.90)	55 (1.15)	0.2347
HPV33	105 (0.97)	0.79–1.17	55 (0.90)	50 (1.05)	0.5077
HPV56	103 (0.95)	0.78–1.15	49 (0.80)	54 (1.13)	0.1006
HPV68	95 (0.87)	0.71–1.07	51 (0.84)	44 (0.92)	0.7167
HPV31	76 (0.70)	0.55–0.88	41 (0.67)	35 (0.73)	0.7990
HPV39	68 (0.63)	0.49–0.80	30 (0.49)	38 (0.79)	0.0620
HPV35	56 (0.51)	0.39–0.67	33 (0.54)	23 (0.48)	0.7641
HPV59	39 (0.36)	0.26–0.50	20 (0.33)	19 (0.40)	0.6604
HPV45	26 (0.24)	0.16–0.36	10 (0.16)	16 (0.33)	0.1071
HPV66	20 (0.18)	0.12–0.29	11 (0.18)	9 (0.19)	1.0000

Data are shown as *n* (%). Analyses in this table were restricted to participants with classifiable institution-level proxy information (*n* = 10,876). Therefore, overall genotype-specific counts in this table may differ slightly from those reported for the full analytic cohort. Percentages were calculated within each institution-level proxy category. The institution-level proxy was derived from the submitting institution and should be interpreted as a proxy for the submitting-site setting rather than verified residence. *p*-values were derived from Pearson’s Chi-square test for all genotype-specific comparisons between the two institution-level proxy categories.

**Table 6:** Prevalence of individual high-risk HPV E6/E7 mRNA genotypes according to department-based clinical-context proxy category.

High-risk HPV genotype	Overall positive, *n* (%)	95% CI	Gynecology-related, *n* (%), (*n* = 6,654)	Opportunistic screening, *n* (%), (*n* = 3,954)	*p*-value
HPV52	603 (5.68)	5.26–6.15	472 (7.09)	131 (3.31)	<0.001
HPV58	285 (2.69)	2.39–3.02	234 (3.52)	51 (1.29)	<0.001
HPV16	218 (2.06)	1.80–2.35	185 (2.78)	33 (0.83)	<0.001
HPV18	125 (1.18)	0.99–1.41	104 (1.56)	21 (0.53)	<0.001
HPV51	108 (1.02)	0.84–1.23	87 (1.31)	21 (0.53)	<0.001
HPV33	104 (0.98)	0.81–1.19	86 (1.29)	18 (0.46)	<0.001
HPV56	99 (0.93)	0.76–1.14	81 (1.22)	18 (0.46)	<0.001
HPV68	94 (0.89)	0.72–1.09	71 (1.07)	23 (0.58)	0.0134
HPV31	70 (0.66)	0.52–0.84	51 (0.77)	19 (0.48)	0.1021
HPV39	64 (0.60)	0.47–0.77	50 (0.75)	14 (0.35)	0.0153
HPV35	52 (0.49)	0.37–0.65	38 (0.57)	14 (0.35)	0.1604
HPV59	36 (0.34)	0.24–0.47	31 (0.47)	5 (0.13)	0.0063
HPV45	27 (0.25)	0.17–0.38	20 (0.30)	7 (0.18)	0.3069
HPV66	18 (0.17)	0.10–0.27	17 (0.26)	1 (0.03)	0.0110

Data are shown as *n* (%). Analyses in this table were restricted to participants with classifiable department-based clinical-context proxy information (*n* = 10,608); therefore, overall genotype-specific counts in this table may differ slightly from those reported for the full analytic cohort. Percentages were calculated within each proxy category. The department-based proxy was derived from the submitting department and should be interpreted as a proxy for testing context rather than the definitive indication for testing. *p*-values were derived from Pearson’s Chi-square test for all genotype-specific comparisons between the two department-based proxy categories.

Categorical variables were compared using Pearson’s chi-square test when expected cell counts were adequate. Fisher’s exact test was used for sparse comparisons with small expected counts, particularly for low-frequency genotype-specific analyses. For genotype-specific comparisons across age groups, regions, or proxy-defined subgroups, *p*-values were interpreted as exploratory and unadjusted.

To quantify associations with HPV E6/E7 mRNA positivity, univariable and multivariable logistic regression models were fitted. The primary multivariable model evaluated factors associated with any high-risk HPV E6/E7 mRNA positivity, including age, geographic region, institution-level proxy category, and department-based clinical-context proxy category as covariates. Secondary exploratory models were fitted for genotype-specific positivity for HPV16, HPV52, and HPV58. Results are reported as odds ratios (ORs) or adjusted odds ratios (aORs) with 95% CIs.

**Figure 2:**
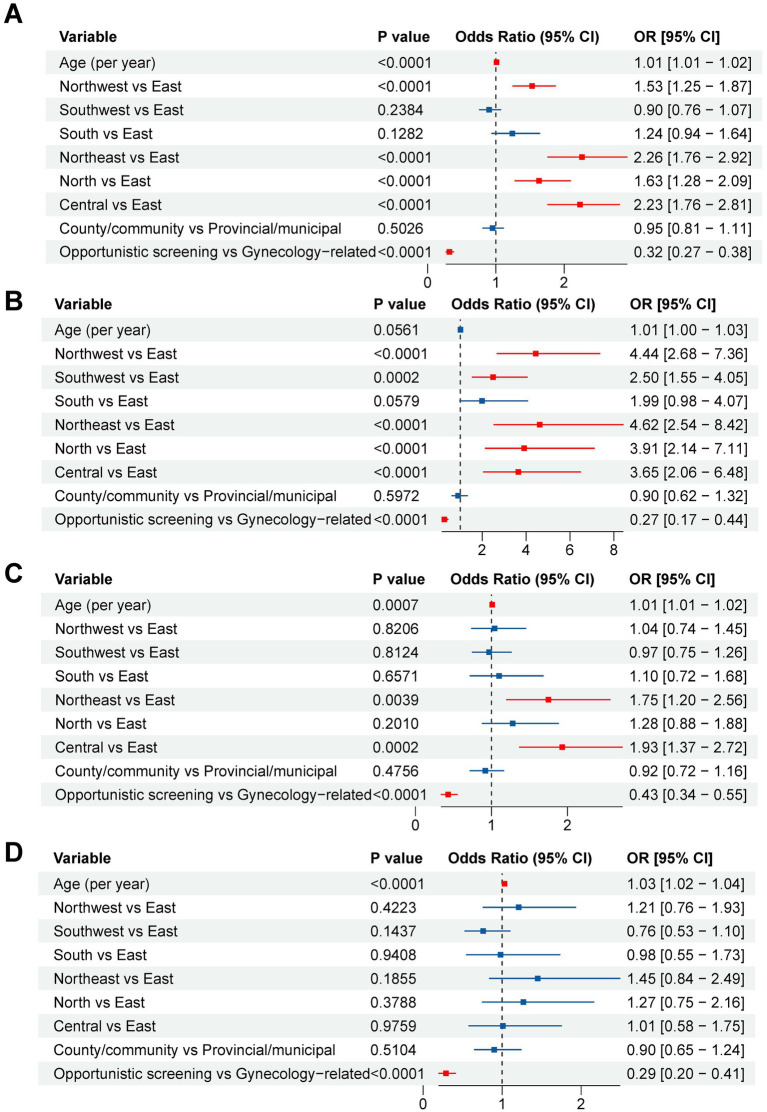
Multivariable logistic regression analyses of factors associated with overall and genotype-specific high-risk HPV E6/E7 mRNA positivity. **(A)** Forest plot showing adjusted odds ratios (aORs) and 95% confidence intervals (CIs) for factors associated with any high-risk HPV E6/E7 mRNA positivity. **(B)** Forest plot showing aORs and 95% CIs for factors associated with HPV16 E6/E7 mRNA positivity. **(C)** Forest plot showing aORs and 95% CIs for factors associated with HPV52 E6/E7 mRNA positivity. **(D)** Forest plot showing aORs and 95% CIs for factors associated with HPV58 E6/E7 mRNA positivity. Models included age, geographic region, institution-level proxy category, and department-based clinical-context proxy category. The dashed vertical line indicates an aORs of 1.0. Reference groups were East region, provincial/municipal-level institution proxy, and gynecology-related clinical attendance proxy. Age was modeled as a continuous variable (per year increase). Points represent aORs and horizontal lines represent 95% confidence intervals. aOR, adjusted odds ratio; CI, confidence interval; HPV, human papillomavirus.

Robust standard errors clustered at the submitting-institution level were employed to address site-level clustering. Multivariable analysis results for any high-risk HPV and the three most prevalent genotypes (HPV16, 52, and 58) are integrated and presented in Figure 2.

For analyses involving institution-level or department-based proxy variables, records with unclassifiable proxy information were excluded from the corresponding subgroup-specific analyses. In addition, a sensitivity analysis restricted to the opportunistic screening proxy subgroup was performed to better approximate screening-context prevalence estimates; these results are presented in the Supplementary material. All statistical tests were two-sided. Statistical analyses were performed using R version 4.3.1.

## Results

### Cohort characteristics and overall high-risk HPV E6/E7 mRNA positivity

The final analytic cohort comprised 11,118 female cervical specimens retrieved from routine clinical testing across seven geographic regions of China (Figure 1). The median age was 42.0 years [interquartile range (IQR), 35.0–51.0 years], and the largest proportion of samples originated from East China (44.3%) (Table 1). Institution-level proxy information was classifiable for 10,876 samples, and department-based clinical-context proxy information was classifiable for 10,608 samples (Table 1). Overall, 1,815 of 11,118 samples were positive for at least one high-risk HPV E6/E7 mRNA genotype, corresponding to an overall positivity rate of 16.32%. Overall positivity differed significantly across age groups, geographic regions, institution-level proxy categories, and department-based clinical-context proxy categories (all overall *p* < 0.001) (Table 2). Positivity was lowest among women aged 41–50 years (14.0%) and increased in older age groups, reaching 20.2% in those aged 51–60 years and 24.5% in those aged >60 years. Across regions, overall positivity ranged from 13.0% in East China to 29.8% in Central China. In addition, positivity was modestly higher in the county/community institution proxy group than in the provincial/municipal-level proxy group (17.7% vs. 15.0%), and markedly higher in the gynecology-related clinical attendance proxy group than in the opportunistic screening proxy group (20.1% vs. 8.6%) (Table 2).

### National and regional distribution of high-risk HPV E6/E7 mRNA genotypes

**Figure 3:**
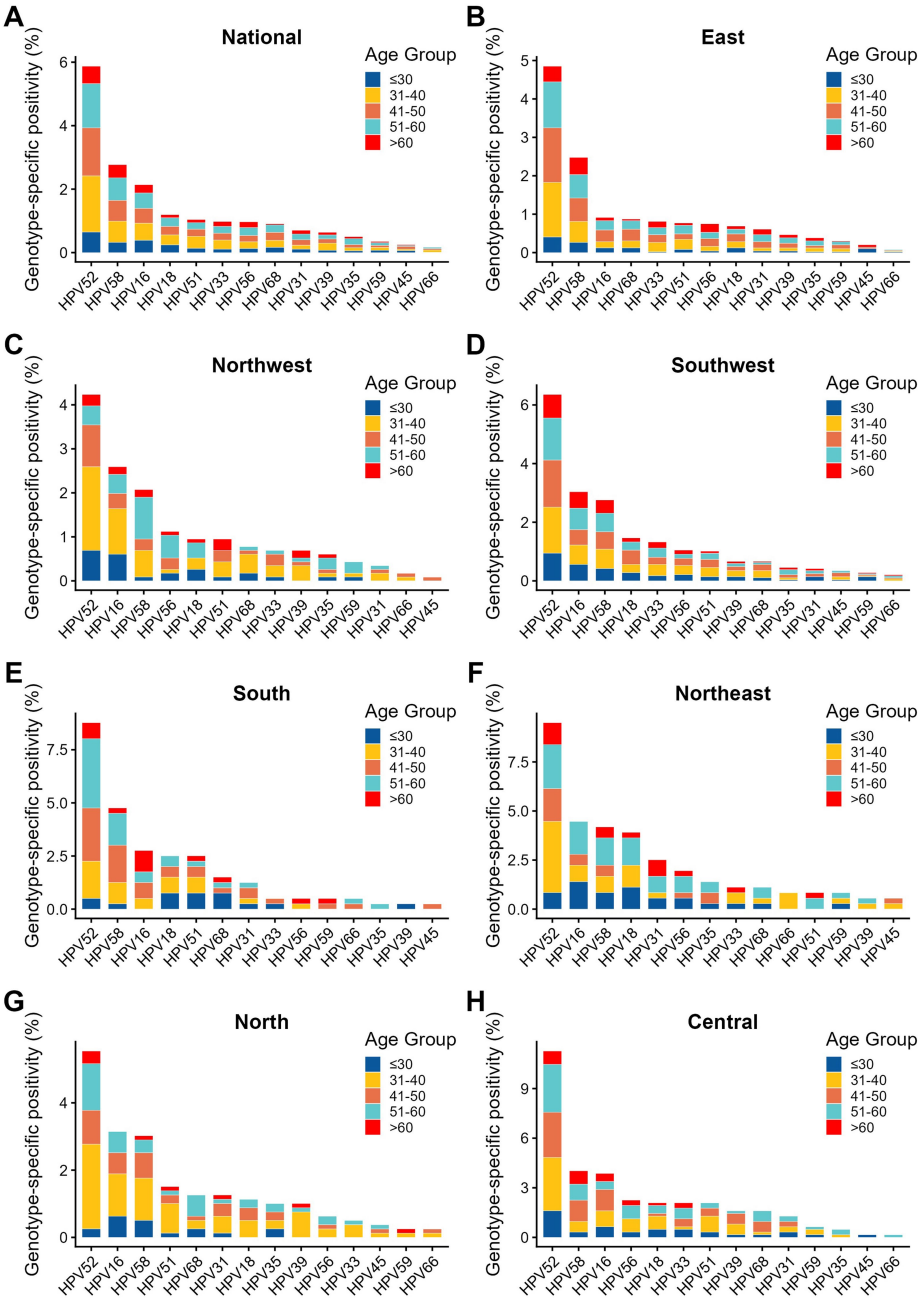
Age- and region-stratified distribution of high-risk HPV E6/E7 mRNA genotypes. **(A)** National profile of genotype-specific high-risk HPV E6/E7 mRNA positivity across the full analytic cohort. The *x*-axis shows the 14 high-risk HPV genotypes, and the *y*-axis shows genotype-specific positivity (%). Bars are displayed as stacked columns stratified by age group (≤30, 31–40, 41–50, 51–60, and >60 years). **(B–H)** Region-specific profiles of genotype-specific high-risk HPV E6/E7 mRNA positivity stratified by age group in East **(B)**, Northwest **(C)**, Southwest **(D)**, South **(E)**, Northeast **(F)**, North **(G)**, and Central China **(H)**. Within each panel, the x-axis represents the 14 high-risk HPV genotypes and the *y*-axis represents genotype-specific positivity (%), with stacked colors indicating the age-group distribution for each genotype. HPV, human papillomavirus.

At the national level, a clear hierarchy of transcriptionally active genotypes was observed, dominated by HPV52 (5.87%), HPV58 (2.77%), HPV16 (2.14%), HPV18 (1.20%), and HPV51 (1.04%) (Table 3, Figure 3A). HPV52 remained the most prevalent genotype across all seven geographic regions (Table 4, Figures 3B–H). Substantial geographic heterogeneity was observed for several genotype-specific positivity rates (Table 4). Across all seven regions, HPV52 consistently ranked as the most prevalent transcriptionally active genotype, followed by varying distributions of HPV58 and HPV16 as the second or third most frequent genotypes (Supplementary Table S3). HPV52 positivity varied from 4.24% in Northwest China to 11.27% in Central China (*p* < 0.001), with similarly elevated levels in Northeast China (9.50%) and South China (8.77%). HPV58 also showed significant regional variation, ranging from 2.07% in Northwest China to 4.76% in South China (*p* = 0.0177). HPV16 displayed particularly marked regional heterogeneity (*p* < 0.001), with the lowest positivity in East China (0.91%) and higher positivity in Northeast China (4.47%), Central China (3.86%), North China (3.15%), and Southwest China (3.04%). Significant regional heterogeneity was also observed for HPV18, HPV51, HPV33, HPV56, HPV31, and HPV66, whereas lower-frequency genotypes such as HPV68, HPV39, HPV35, HPV59, and HPV45 showed no statistically significant regional differences (Table 4). Overall, these findings indicate that while HPV52, HPV58, and HPV16 dominate the active high-risk HPV landscape nationally, their relative distribution varies considerably across regions (Table 4, Figure 3).

### Age-specific patterns of genotype-specific positivity

Age-stratified analyses revealed significant heterogeneity for several major genotypes (Table 3). HPV52 positivity increased in older age groups, rising from 4.93% in women aged ≤30 years to 7.27% in those aged 51–60 years and 7.73% in those aged >60 years (*p* = 0.0014). HPV58 showed an even more pronounced age gradient, increasing from 2.47% in women aged ≤30 years to 5.83% in those aged >60 years (*p* < 0.001). HPV16 exhibited a bimodal pattern, with relatively higher positivity in the youngest (2.95%) and oldest (3.68%) age groups and lower positivity in the intermediate age groups (*p* < 0.001). Additional age-related heterogeneity was observed for HPV33, HPV56, HPV31, and HPV35, whereas HPV18 showed only borderline significance and several other genotypes, including HPV51, HPV68, HPV39, HPV59, HPV45, and HPV66, showed no statistically significant age-related differences (Table 3). The regional stacked-bar profiles further showed that HPV52 remained the leading genotype across age strata in all seven regions, while the relative age composition of genotype-specific positivity varied across geographic settings (Figures 3B–H). Taken together, these data indicate that genotype-specific positivity is shaped by both age and geography, with particularly strong age dependence for HPV52, HPV58, and HPV16 (Table 3, Figure 3).

### Proxy-defined differences in genotype-specific positivity

**Figure 4:**
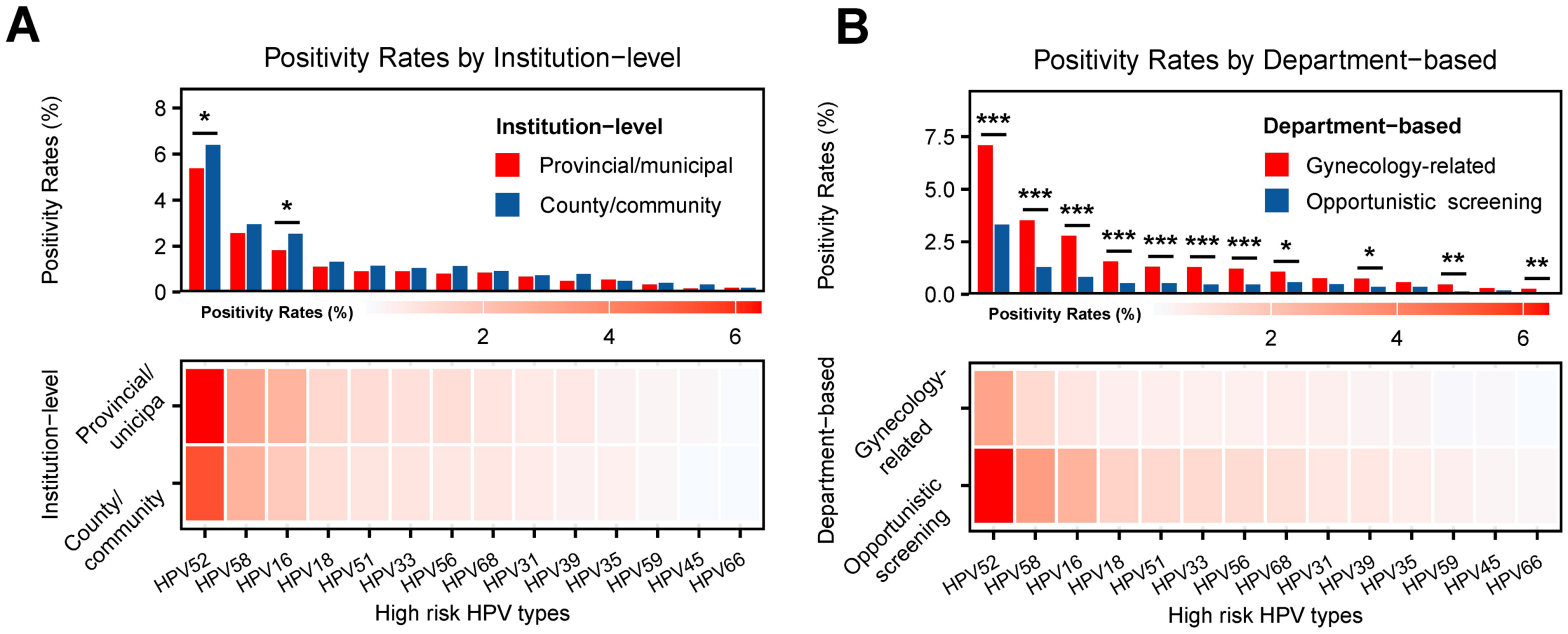
Genotype-specific high-risk HPV E6/E7 mRNA positivity according to institution-level and department-based proxy categories. **(A)** Prevalence of specific high-risk HPV types by institution-level proxy. **(B)** Prevalence of specific high-risk HPV types by department-based proxy. Bar charts: The bars represent the prevalence (%) of each specific genotype within each respective category. The institution-level proxy categorized participants into “Provincial/municipal” level hospitals (*N* = 6,096) and “County/community” facilities (*N* = 4,780). The department-based proxy categorized participants into “Gynecology-related” departments (*N* = 6,654) and “Opportunistic screening” participants (*N* = 3,954). The *N* values for percentage denominators were calculated based on available data for each specific characteristic. Heatmaps: The heatmaps below the bar charts provide a visual representation of genotype prevalence across the categories using color intensity, with darker red indicating higher positivity rates and lighter colors representing lower rates. Genotypes on the *x*-axis are ordered from most prevalent to least prevalent. The heatmaps include a color scale bar indicating the magnitude of genotype-specific positivity, with darker red representing higher positivity rates and lighter shades representing lower positivity rates. Statistical significance: *p*-values were calculated to compare the prevalence of each genotype between the two groups within each panel (e.g., in Panel A, provincial/municipal vs. county/community). Comparisons with no stars have a *p* > 0.05 (not statistically significant). Significance is denoted as follows: *p < 0.05; **p < 0.01; ***p < 0.001.

To better characterize source-population heterogeneity, genotype-specific positivity was further compared across institution-level and department-based proxy categories. Among samples with classifiable institution-level proxy information, modest but statistically significant differences were observed for selected genotypes (Table 5, Figure 4A). HPV52 positivity was higher in the county/community institution proxy group than in the provincial/municipal-level proxy group (6.40% vs. 5.38%, *p* = 0.0268), and a similar pattern was observed for HPV16 (2.53% vs. 1.82%, *p* = 0.0132). HPV39 also showed a small difference between the two institution-level proxy categories (0.79% vs. 0.49%, *p* = 0.0620), whereas most other genotypes did not differ significantly (Table 5).

Beyond individual genotype prevalence, we further characterized the complexity of these detections by examining infection patterns (single vs. multiple types) across the proxy-defined groups. While single-type detections predominated across all institutional settings, the proportion of specific co-detection combinations and the overall distribution of multi-type patterns among provincial/municipal-level and county/community-level institution proxies are detailed in Supplementary Table S4. Similarly, when analyzed by clinical attendance source, the breakdown of single, double, and higher-order detection patterns—including the most frequent genotype combinations—revealed distinct source-population heterogeneity between women from gynecology-related departments and those undergoing opportunistic screening (Supplementary Table S5).

By contrast, more pronounced differences were observed across department-based clinical-context proxy categories (Table 6, Figure 4B). Genotype-specific positivity was consistently higher in the gynecology-related clinical attendance proxy group than in the opportunistic screening proxy group for most major genotypes, including HPV52 (7.09% vs. 3.31%), HPV58 (3.52% vs. 1.29%), and HPV16 (2.78% vs. 0.83%) (all *p* < 0.001). Similar patterns were observed for HPV18, HPV51, HPV33, HPV56, HPV68, HPV39, HPV59, and HPV66, whereas HPV31, HPV35, and HPV45 did not differ significantly between the two proxy-defined clinical contexts (Table 6). These comparisons support substantial heterogeneity in active HPV genotype distribution according to proxy-defined testing setting and clinical context (Figure 4).

### Patterns of multi-type positivity and descriptive co-detection structure

Among the 1,815 high-risk HPV E6/E7 mRNA-positive samples, single-type positivity predominated, accounting for 88.2% of positive samples (1,601/1,815), whereas 10.5% (190/1,815) showed exclusive double-type positivity, 1.1% (20/1,815) showed triple-type positivity, and 0.2% (4/1,815) showed quadruple-or-higher positivity (Supplementary Table S6). The proportion of multi-type positivity varied across regions and was highest in Northeast China (18.0%) and Southwest China (16.1%), followed by Central China (12.4%) and North China (12.2%), while lower proportions were observed in East China (8.6%), Northwest China (9.0%), and South China (8.2%) (Supplementary Table S6).

**Figure 5:**
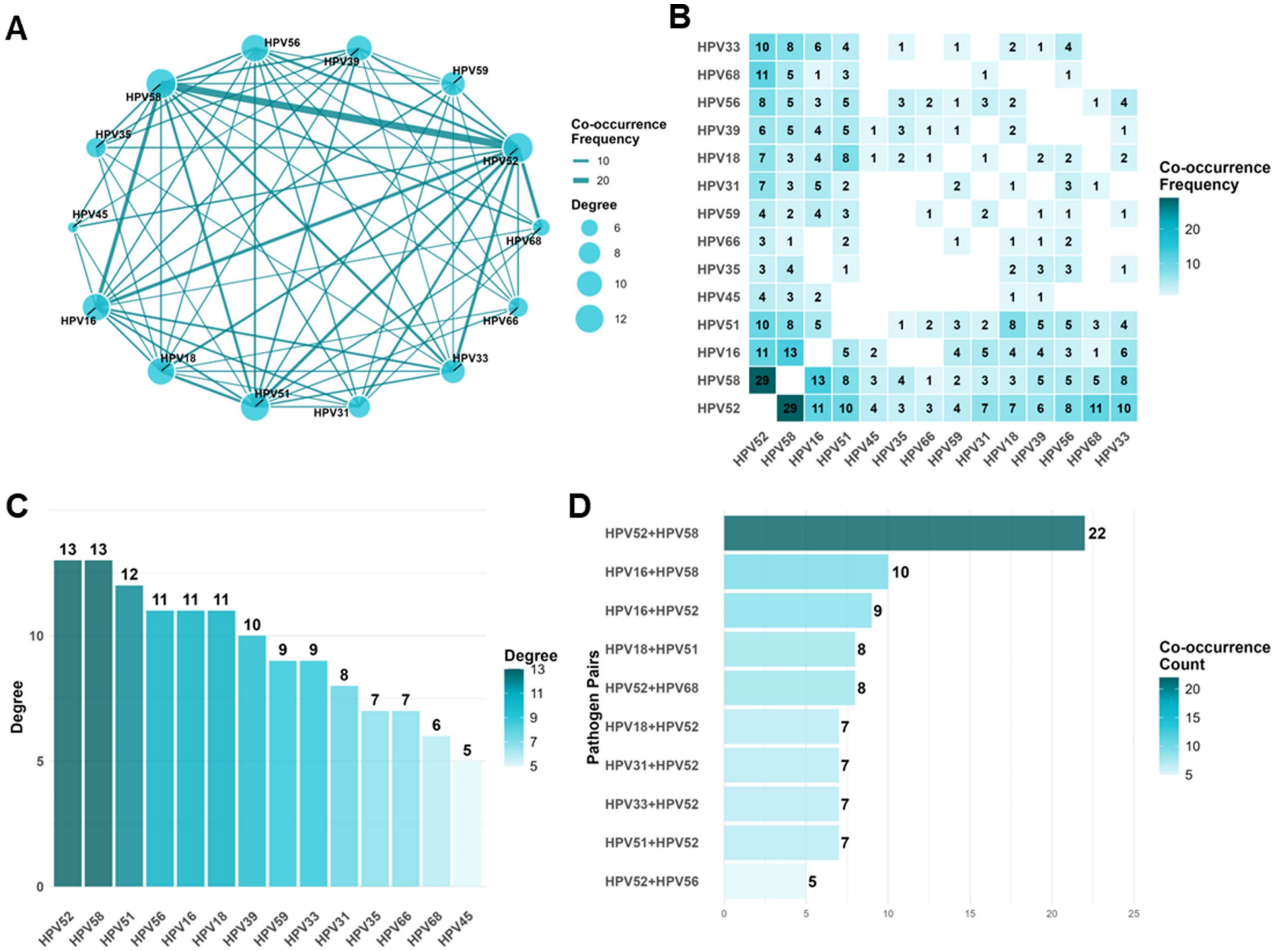
Network-based summary of co-detection patterns among high-risk HPV E6/E7 mRNA genotypes. **(A)** Undirected weighted network showing pairwise co-detection patterns among the 14 high-risk HPV E6/E7 mRNA genotypes in multi-type positive samples. Each node represents one genotype, node size is proportional to its degree (the number of distinct genotypes with which it co-occurred), and edge thickness is proportional to the pairwise co-detection frequency. **(B)** Heatmap of pairwise co-detection frequencies among high-risk HPV genotypes. Each cell indicates the number of samples in which the corresponding genotype pair was detected together among multi-type positive samples. Darker shading indicates higher co-detection frequency. **(C)** Degree distribution of the genotype co-detection network. Bars rank genotypes according to degree, reflecting the breadth of their pairwise co-detection with other high-risk HPV genotypes. **(D)** Most frequent exclusive dual-genotype combinations. Counts shown in this panel are restricted to samples positive for exactly two genotypes and therefore exclude samples with triple or higher-order co-detection. HPV, human papillomavirus.

In the descriptive co-detection network, HPV52 occupied the most central position and showed co-detection with all other high-risk genotypes analyzed (degree = 13), while HPV58 and HPV16 also exhibited broad connectivity (Figures 5A–C). Across all multi-type positive samples, the HPV52–HPV58 pair had the highest pairwise co-detection frequency (*n* = 29) (Figures 5A,B). When analysis was restricted to samples positive for exactly two genotypes, HPV52 + HPV58 remained the most frequent exclusive dual-genotype combination (*n* = 22), followed by HPV16 + HPV58 (*n* = 10) and HPV16 + HPV52 (*n* = 9) (Figure 5D, Supplementary Table S6). Triple and higher-order combinations were uncommon; the most frequent triple combinations were HPV16 + HPV33 + HPV58 and HPV52 + HPV58 + HPV68, each detected twice (Supplementary Table S6). Overall, these findings indicate a descriptive co-detection structure centered on HPV52 and HPV58, with HPV16 acting as a frequent co-detected partner (Figure 5).

### Multivariable analyses of factors associated with overall and genotype-specific positivity

To account for potential confounding by age, region, institution-level proxy, and department-based clinical-context proxy, multivariable logistic regression analyses were performed with robust standard errors clustered at the submitting-institution level (Figure 2A). For any high-risk HPV E6/E7 mRNA positivity, older age was associated with a modest increase in aOR [adjusted OR (aOR) per year, 1.01; 95% CI, 1.01–1.02; *p* < 0.001]. Compared with East China, significantly higher aOR were observed in Northwest China (aOR, 1.53; 95% CI, 1.25–1.87), Northeast China (aOR, 2.26; 95% CI, 1.76–2.92), North China (aOR, 1.63; 95% CI, 1.28–2.09), and Central China (aOR, 2.23; 95% CI, 1.76–2.81), whereas Southwest and South China did not remain significantly different after adjustment. The institution-level proxy category was not independently associated with overall positivity, but the opportunistic screening proxy group had substantially lower aOR of positivity than the gynecology-related clinical attendance proxy group (aOR, 0.32; 95% CI, 0.27–0.38; *p* < 0.001) (Figure 2A).

Genotype-specific multivariable models further showed distinct patterns (Figures 2B–D). For HPV16 positivity, strong regional heterogeneity persisted after adjustment, with significantly higher aOR in Northwest, Southwest, Northeast, North, and Central China relative to East China, whereas age and institution-level proxy were not independently associated with HPV16 positivity (Figure 2B). For HPV52 positivity, older age remained independently associated with positivity (aOR per year, 1.01; 95% CI, 1.01–1.02; *p* = 0.0007), and significant regional associations remained for Northeast China (aOR, 1.75; 95% CI, 1.20–2.56) and Central China (aOR, 1.93; 95% CI, 1.37–2.72), but not for the institution-level proxy (Figure 2C). For HPV58 positivity, age remained positively associated with positivity (aOR per year, 1.03; 95% CI, 1.02–1.04; *p* < 0.001), whereas none of the regional comparisons or the institution-level proxy category remained significant after adjustment (Figure 2D). Across all three genotype-specific models, the opportunistic screening proxy group consistently showed substantially lower aOR of positivity than the gynecology-related clinical attendance proxy group (Figures 2B–D).

## Discussion

This nationwide study provides, to our knowledge, the first large-scale description of HPV E6/E7 mRNA positivity across multiple geographic regions of China. We found that transcriptionally active high-risk HPV detection in this real-world testing cohort was dominated by HPV52, HPV58, and HPV16, with substantial heterogeneity across age groups, geographic regions, and proxy-defined clinical testing contexts. In addition, multi-type positivity accounted for a minority of positive samples and showed a descriptive co-detection structure centered on HPV52 and HPV58.

Our mRNA-based findings are broadly consistent with prior DNA-based epidemiologic studies showing that HPV52 and HPV58, together with HPV16, are among the most prevalent high-risk genotypes in China (17). The present study extends this literature by focusing on E6/E7 mRNA positivity, which reflects viral oncogene transcription rather than viral presence alone. In this respect, our findings suggest that the same genotypes that dominate DNA prevalence in China also account for a large proportion of transcriptionally active infections. This observation may be relevant for risk stratification, although our dataset does not include cytologic or histologic endpoints and therefore cannot directly quantify genotype-specific progression risk.

The age- and region-related patterns observed here also add to current understanding of HPV epidemiology in China. Significant age-related heterogeneity was observed for several major genotypes, particularly HPV52, HPV58, and HPV16, and marked regional variation was evident for overall positivity as well as for key genotype-specific distributions. These findings are biologically plausible and are broadly compatible with prior concepts related to age-dependent exposure, persistence, and possible reactivation (18–20). However, the mechanisms underlying these patterns cannot be determined from our cross-sectional data and should be regarded as hypothesis-generating rather than causal. Likewise, the higher overall positivity observed in Central, Northeast, and some other regions may reflect a combination of population structure, healthcare access, testing patterns, background genotype ecology, and other unmeasured factors.

To further characterize heterogeneity in the study population, we evaluated proxy variables derived from routine laboratory submission data. These analyses showed that overall and genotype-specific positivity differed not only across age groups and geographic regions, but also across proxy-defined clinical testing contexts. In particular, samples from the opportunistic screening proxy group consistently showed lower odds of positivity than those from the gynecology-related clinical attendance proxy group in both overall and genotype-specific multivariable models. This pattern likely reflects differences in underlying pre-test probability and source-population composition. At the same time, these proxy variables should be interpreted cautiously. The institution-level proxy reflects the administrative level and service setting of the submitting institution rather than the participant’s verified place of residence, and the department-based proxy reflects clinical testing context rather than the definitive indication for testing. Accordingly, our data are best interpreted as describing transcriptionally active HPV infection in a large real-world testing population rather than in a strictly population-based screening cohort.

Our co-detection analyses further showed that HPV52 and HPV58 occupied central positions in the descriptive co-detection network, with HPV16 as another frequent co-detected partner. The HPV52 plus HPV58 combination was the most common exclusive dual-genotype pair. These observations may be relevant for future studies of persistence, clearance, and genotype interaction. However, the network presented here is descriptive, and our data do not allow inference regarding biological synergy, competitive interaction, or differential lesion risk associated with specific co-detection patterns. Longitudinal cohorts with outcome linkage will be required to determine whether particular multi-type combinations carry distinct clinical significance.

For screening, E6/E7 mRNA provides higher clinical specificity for progressive disease by capturing oncogene transcription, supporting its integration into risk-stratified algorithms to reduce unnecessary colposcopy and treatment while preserving safety (21–23). Our findings therefore may inform further evaluation of activity-based or genotype-aware triage strategies, particularly in settings with high burdens of HPV52 and HPV58. Similarly, the active genotype spectrum observed here is broadly compatible with current nonavalent vaccine coverage, since HPV16, 52, and 58 are all included in the 9-valent formulation. Recent reviews and vaccine-impact studies also support continued genotype-specific surveillance to monitor real-world changes in vaccine-targeted HPV distributions over time (22, 24). However, our study was not designed to evaluate vaccine effectiveness, and implications for vaccination should therefore be interpreted cautiously. Rather than supporting direct policy conclusions, our data provide baseline information that may be useful for future surveillance of transcriptionally active infections and for assessing whether genotype distributions shift over time or differ across regions.

This study has several strengths. It includes a large sample size, broad geographic coverage, assessment of 14 high-risk genotypes, and a focus on HPV E6/E7 mRNA rather than DNA alone. The analyses also incorporated proxy measures of source-population composition and multivariable regression with institution-level cluster-robust standard errors, strengthening the evaluation of regional and contextual heterogeneity. Several limitations should also be acknowledged. First, the retrospective cross-sectional design precludes temporal or causal inference. Second, cytology, histology, vaccination history, and many behavioral or clinical covariates were not available, limiting direct interpretation of clinical risk and leaving potential residual confounding. Third, the proxy variables used for institution level and clinical testing context are informative but imperfect and should not be interpreted as direct measures of residence, organized screening participation, or diagnostic indication. Finally, because histologic endpoints were unavailable, this study cannot determine genotype-specific progression risk or directly evaluate the clinical performance of E6/E7 mRNA-based management strategies.

In conclusion, this nationwide study shows that transcriptionally active high-risk HPV infection in China is characterized by dominance of HPV52, HPV58, and HPV16, together with substantial heterogeneity across age groups, regions, and proxy-defined testing contexts. These data provide a national overview of HPV E6/E7 mRNA positivity in a large real-world testing population and may serve as a foundation for future prospective studies linking transcriptionally active infection to cytologic and histologic outcomes, as well as for regionally stratified surveillance of HPV prevention programs in China.

## Data Availability

The datasets analyzed in this study are not publicly available because they contain de-identified retrospective clinical laboratory records that are subject to institutional data governance and privacy restrictions. Requests to access these datasets should be directed to Jingna Sun, 57600491@hebmu.edu.cn.
